# Immunolocalization of the Tumor-Sensitive Calmodulin-Like Protein CALML3 in Normal Human Skin and Hyperproliferative Skin Disorders

**DOI:** 10.1371/journal.pone.0062347

**Published:** 2013-04-18

**Authors:** Richard D. Bennett, Mark R. Pittelkow, Emanuel E. Strehler

**Affiliations:** 1 Department of Biochemistry and Molecular Biology, Mayo Clinic College of Medicine, Rochester, Minnesota, United States of America; 2 Department of Cell Biology and Genetics Program, Graduate School, Mayo Clinic College of Medicine, Rochester, Minnesota, United States of America; 3 Department of Dermatology, Mayo Clinic College of Medicine, Rochester, Minnesota, United States of America; Università degli Studi di Milano, Italy

## Abstract

**Background and Objective:**

Calmodulin-like protein CALML3 is an epithelial-specific protein regulated during keratinocyte differentiation *in vitro*. CALML3 expression is downregulated in breast cancers and transformed cell lines making it an attractive marker for tumor formation. The objective of this study was to survey CALML3 localization in normal epidermis and in hyperproliferative skin diseases including actinic keratosis, squamous and basal cell carcinoma as well as verruca and psoriasis and to compare CALML3 immunoreactivity with the proliferation marker Ki-67.

**Methods:**

Paraffin-embedded tissue sections from normal human skin and hyperproliferative skin disorders were examined by immunohistochemistry and analyzed for localization and expression of CALML3 and Ki-67.

**Results:**

CALML3 was strongly expressed in differentiating layers of normal skin, staining the periphery in suprabasal cells and exhibiting nuclear localization in the stratum granulosum. CALML3 nuclear localization was inversely correlated to Ki-67 staining in each disease, indicating that CALML3 nuclear presence is related to terminal cell differentiation and postmitotic state.

**Conclusions:**

Increased CALML3 expression in suprabasal layers is characteristic for differentiating keratinocytes in normal epidermis, and nuclear expression of CALML3 inversely correlates with expression of the proliferation marker Ki-67. This suggests that CALML3 is a useful marker for normal and benign hyperplastic epidermal development, whereas the loss of nuclear CALML3 indicates progression to a proliferative and potentially malignant phenotype.

## Introduction

Calmodulin-like-protein CALML3 (also known as CLP) is an epithelial-specific calcium-sensing protein that is highly expressed in tissues such as breast, prostate, and skin [1], [2]. Although the function of CALML3 has not yet been fully elucidated, recent work has shown that it is a regulator of the unconventional myosin-10 [3], [4], which may be important in cell adhesion and motility [5]–[7]. We have previously reported that CALML3 is regulated in human keratinocytes during differentiation and increases in abundance from the suprabasal to keratinized layers [8], whereas it is downregulated in breast tumors and in transformed cells in culture [2], [9]. This interesting pattern of expression makes CALML3 a potentially attractive marker of progression from a differentiated, post-mitotic to an undifferentiated, proliferative, and potentially tumorigenic state in stratified epithelial tissues.

Skin hyperproliferative disorders display a wide range in source, severity, and etiology. Actinic keratosis (AK) is a common pre-cancerous skin lesion that is of concern because of its potential to develop into squamous cell carcinoma. Basal cell carcinomas (BCC) and squamous cell carcinomas (SCC) are the most common non-melanoma skin cancers with over 2 million cases diagnosed each year in the US alone (American Cancer Society, Reference Information on Skin Cancer, last review 06/27/2011, http://www.cancer.org/). Some remain localized and well differentiated, while others can become invasive and exhibit a less differentiated and a more proliferative phenotype. Since CALML3 is expressed in a well-defined pattern in normal skin, aberrant expression can be easily visualized by immunohistochemical staining of biopsy sections. Any loss or change of immuno-reactivity may indicate a change away from normal development towards a potentially invasive lesion and indicate a specific diagnosis or course of treatment.

The aims of this study were to characterize the expression and localization of CALML3 in skin hyperproliferative disorders and correlate these data with the morphological changes that accompany transition from benign/pre-cancerous to cancerous hyperplasia using a CALML3-specific antibody previously developed in our laboratory [2]. We further compare the immunoreactivity of CALML3 with staining for Ki-67. Ki-67 is a well-established marker for proliferating cells that localizes specifically to the nucleus though its function is not completely clear [10]. Here we focus on CALML3 expression in actinic keratosis (AK), squamous cell carcinoma (SCC), and basal cell carcinoma (BCC). Localization of CALML3 in acanthoma, verruca (warts) and the inflammatory hyperproliferative skin disease psoriasis is also examined and discussed.

## Materials and Methods

### Ethics Statement

This study was approved by the Mayo Foundation Institutional Review Board as protocol PR-06-003018, as well as by the Mayo Rochester Biospecimens Subcommittee. Because the study involves immunostaining of clinically obtained and archived paraffin-embedded tissue specimens, the IRB approved waiver of specific informed consent in accordance with 45 CFR 46.116, item 5, and waiver of HIPAA authorization in accordance with applicable HIPAA regulations. The IRB committee determined that this study constitutes minimal risk research and therefore was eligible for expedited review.

### Tissue Samples

Formalin-fixed, paraffin-embedded tissue specimens of normal skin and of skin disease biopsies were obtained from the Mayo Clinic Tissue Registry following an IRB approved protocol (PR-06-003018) permitting the use of 50 patient samples. Blocks from 5 normal skin samples (normal margins of excisions for benign lesions of various sites) and 41 skin disease specimens (6 actinic keratosis, 5 basal cell carcinoma, 9 squamous cell carcinoma, 6 acanthoma, 5 verruca, 5 ichthyosis, 5 psoriasis) were selected based on the pathology reports. Blocks were sectioned at 5-µm thickness following standard protocols in the Mayo Clinic Histopathology core facility and sections were subjected to standard hematoxylin and eosin (H&E) staining or immunohistochemistry (IHC).

### Antibodies

Affinity-purified polyclonal rabbit anti-human CALML3 antibody TG7 has been characterized earlier [2]. The mouse monoclonal anti-human Ki-67 antibody (clone mib-1, catalog no. M7240) was purchased from DAKO (Carpinteria, CA).

### Immunohistochemistry

Staining for Ki-67 expression was performed in the Mayo Clinic Tissue and Cell Molecular Analysis (TACMA) Laboratory following standard IHC protocols for epitope retrieval and staining. Briefly, formalin-fixed, paraffin-embedded samples were deparaffinized, re-hydrated, and rinsed well in distilled water. Slides were incubated in preheated 1 mM EDTA (pH 8.0) retrieval buffer in a vegetable steamer for 30 minutes and then cooled in the buffer for 5 min followed by a 5 min rinse in running distilled water. Slides were then stained using a DAKO auto-stainer (at room temperature). Sections were incubated with 3% H_2_O_2_ in ethanol for 5 min, rinsed with TBST wash buffer, then incubated in 1∶100 anti-Ki-67 for 30 min. After rinsing with TBST, the slides were incubated with labeled polymer EnVision+ Dual Link System-HRP (DAKO, Carpenteria, CA) for 15 min. Slides were washed again with TBST and developed in diaminobenzidine (DAB+) for 10 min, counterstained with Modified Schmidts' Hematoxylin, rinsed in tap water, dehydrated and coverslipped.

Detection of CALML3 expression was similarly performed in the Mayo Clinic TACMA Laboratory following the above standard protocols for epitope retrieval and IHC staining as described [2].

### Data Analysis

Slides were examined and photographed with an Olympus DP71 camera mounted on an Olympus BX40 microscope. Two observers (RDB and MRP) independently assessed the cell morphology, CALML3 staining intensity (compared to normal positive controls) and cellular localization (cytoplasm, membrane, nucleus), and Ki-67 expression.

## Results

### CALML3 shows characteristic localization pattern in normal skin

**Figure 1:**
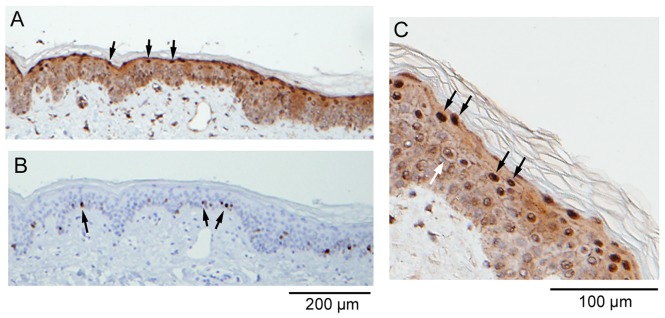
CALML3 expression and localization in normal human skin. Formalin-fixed paraffin-embedded normal skin serial sections were stained for CALML3 (A and C) and Ki-67 (B). Note the polarized expression of CALML3 throughout the epidermis with strong nuclear staining in the upper granular cell layers (black arrows in A and C). CALML3 also stains the cell periphery as seen in the higher magnification image in panel C (white arrow). By contrast, Ki-67 labeling is sparse and confined to the basal cell layer (arrows in B). The images shown are representative of all normal human skin samples analyzed.

In agreement with previous reports [8], [11], CALML3 immunostaining was detected in all layers of the epidermis, with increasingly intense staining from the suprabasal to the granular layers (Fig. 1). Besides diffuse cytoplasmic staining CALML3 shows distinct localization at the cell periphery, especially in the spinous and granular layers. In the uppermost epidermal layers CALML3 exhibits characteristic and strong nuclear staining (Fig. 1C). Accessory organs of the skin such as sebaceous glands and hair follicles also exhibit intense CALML3 staining (not shown but see, e.g., Fig. 2 in ref [8]). In contrast, Ki-67 staining is weak in normal skin with limited staining of the basal layer representing the proliferating keratinocytes (Fig. 1B).

**Figure 2:**
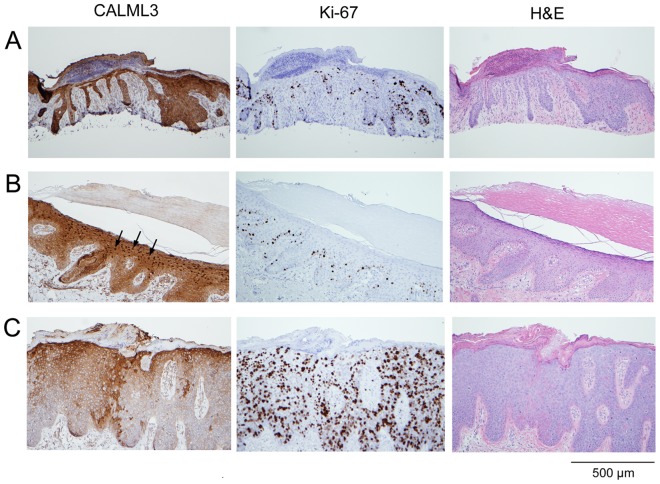
Variable CALML3 and Ki-67 expression in actinic keratosis. Different AK specimens were stained for CALML3, Ki-67, and H&E as indicated. (A) Example of an AK with moderate proliferation and strong CALML3 immunoreactivity but loss of CALML3 nuclear reactivity. (B) AK with mild hyperproliferation confined to the basal layer and robust CALML3 immunoreactivity in the basal and suprabasal layers with nuclear accumulation in the thickened non-proliferative layers (arrows in CALML3-stained panel). (C) Highly proliferative AK with reduced and patchy CALML3 staining in the basal and suprabasal layers and loss of CALML3 nuclear immunoreactivity. This lesion may represent a transition to SCC (see Fig. 3). The images in panels A, B, and C are representative of 2, 3, and 1 of the 6 samples analyzed, respectively.

### Decreased CALML3 staining and absence of nuclear CALML3 expression in non-melanoma skin cancer

To determine the expression and localization of CALML3 in non-melanoma skin cancer, we stained serial sections of human skin tissue samples for CALML3 and the nuclear proliferation marker Ki67. The tissue samples included actinic keratosis (AK), which is often considered a “pre-cancerous” lesion that may eventually develop into invasive squamous cell carcinoma (SCC), as well as specimens of SCC and basal cell carcinoma (BCC). Samples from at least 5 different patients were analyzed for each condition to obtain a representative assessment of the CALML3 expression pattern.

AKs typically appear as rough, scaly patches or as sores or crusts and show a variety of morphologies upon microscopic evaluation ranging from normal to highly atypical epithelium. Fig. 2A shows an AK lesion with relatively sparse Ki-67 staining and high levels of cytoplasmic CALML3 expression but absence of CALML3 nuclear staining, The two extremes of the pattern seen in AKs are illustrated in Fig. 2B and 2C. Figure 2B shows a relatively “normal” sample with mild hyper-proliferation confined to the basal layers (as indicated by the Ki-67 staining) and abundant cytoplasmic CALML3 staining as well as robust CALML3 nuclear staining in the upper layers of the lesion. In contrast, Fig. 2C shows a highly proliferating AK with abundant, scattered Ki-67 staining but patchy CAML3 staining with reduced expression through the basal and suprabasal layers and a loss of nuclear staining in the upper layers of the lesion. Indeed, based on the staining pattern, the lesion shown in Fig. 2C may represent progression to an *in situ* SCC although it was originally identified as AK in the pathology report.

**Figure 3:**
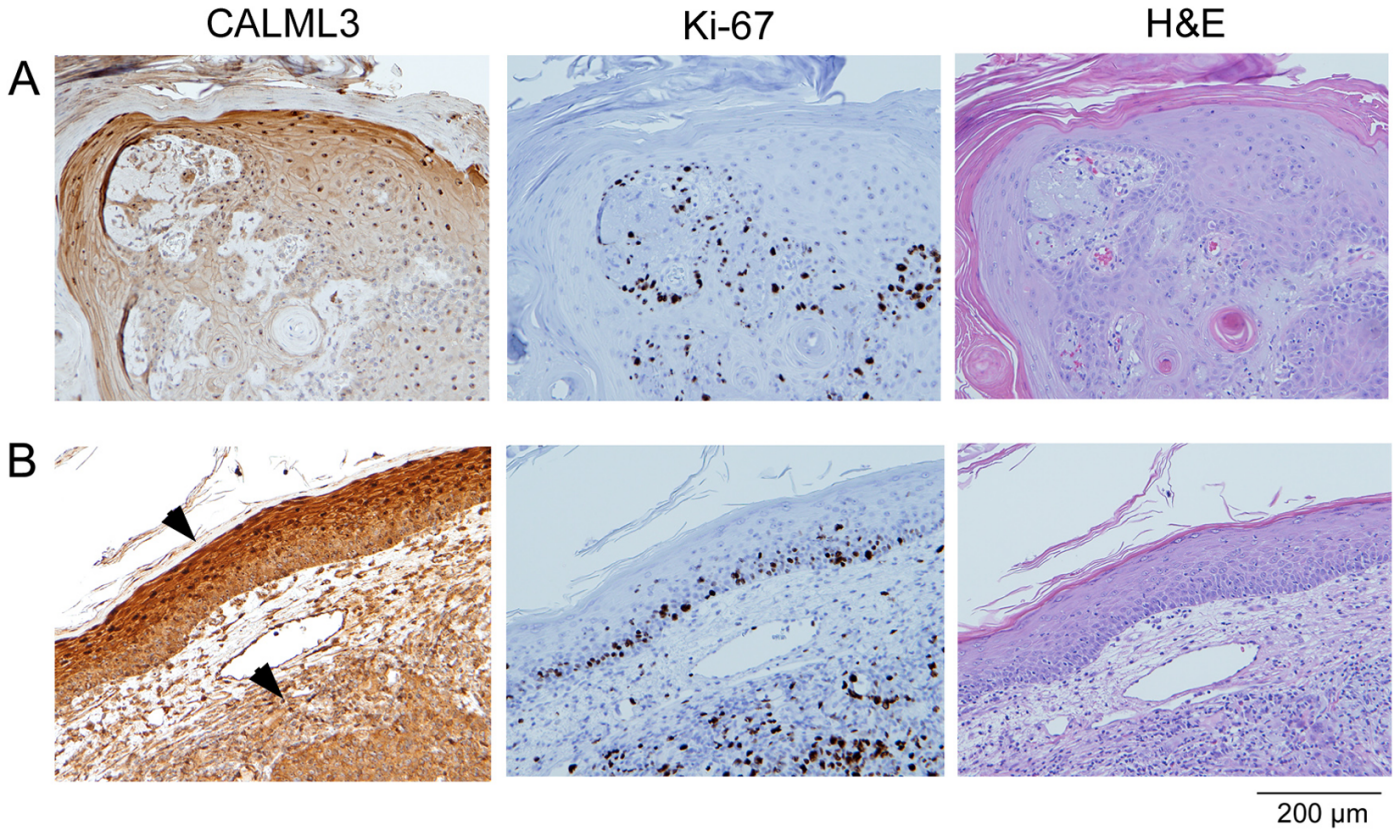
CALML3 in squamous cell carcinoma. SCC samples were serially sectioned and stained for CALML3, Ki-67, and H&E as indicated. (A) Mature, low-grade tumor with regions of proliferation surrounded by differentiated cells expressing cytoplasmic and nuclear CALML3. Note the inverse relationship between CALML3 and Ki-67 staining. (B) SCC with relatively normal but thickened overlying epidermis showing intense CALML3 staining with nuclear localization (upper arrowhead) and underlying tumor with decreased CALML3 staining and loss of nuclear immunolocalization (lower arrowhead). The images shown in panels A and B are each representative of about half of the samples analyzed.

Examples of CALML3 staining in SCC are shown in Figure 3. Fig. 3A shows a lesion with the proliferative tumor surrounded by differentiated cells expressing cytoplasmic and nuclear CALML3, whereas Fig. 3B shows a tumor with overlying normal epidermis and corresponding CALML3 staining of the supra-basal layers. The underlying Ki-67-positive tumor shows reduced CALML3 immunoreactivity and loss of CALML3 nuclear staining. In all samples analyzed, there was an inverse relationship between Ki-67 and CALML3 staining, where CALML3 was consistently reduced with loss of nuclear localization in tumor regions with abundant Ki-67 staining.

**Figure 4:**
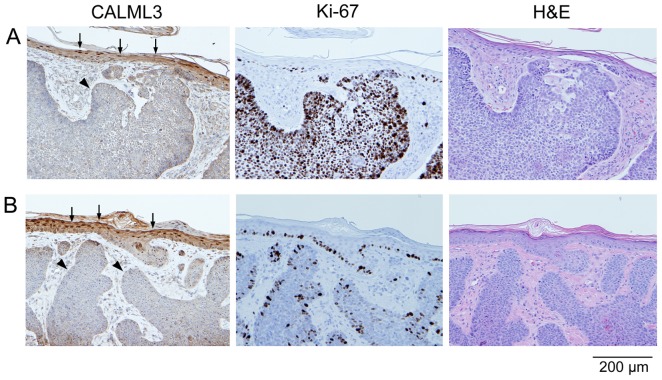
CALML3 in basal cell carcinoma. Serial sections were stained for CALML3, Ki-67, and H&E as indicated. (A) Highly proliferative BCC with strong CALML3 down-regulation in the tumor (arrowhead) but normal CALML3 expression including nuclear localization in the overlying epidermis (arrows). (B) Mildly proliferative BCC with loss of CALML3 expression in the tumor (arrowheads) and normal CALML3 staining in the overlying epidermis (arrows). The images in panels A and B are representative of 3 and 2 of the 5 samples analyzed, respectively.

BCC samples stained for CALML3 showed relatively normal expression and localization in the overlying epidermal layers (Fig. 4). The underlying tumors (Fig. 4A,B) always showed reduced CALML3 immunoreactivity especially in proliferating areas that stained intensely for Ki-67. As observed in SCC, all samples showed a reduction or absence of CALML3 nuclear staining in the tumors.

### CALML3 immunolocalization in psoriasis, verruca, and ichthyosis

**Figure 5:**
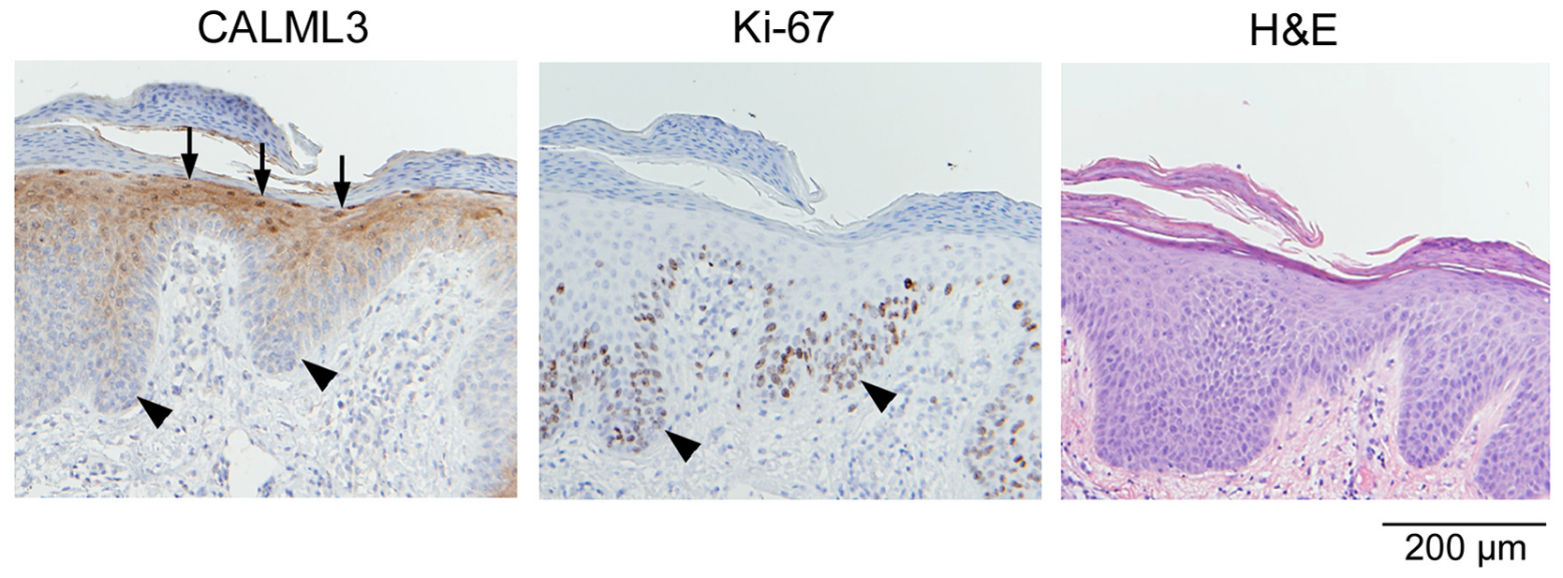
CALML3 and Ki-67 expression in psoriasis. Serial sections of a psoriasis biopsy were stained for CALML3, Ki-67, and H&E as indicated. The example shown is from a highly proliferating lesion. Note the abundant staining for Ki-67 and the reduced CALML3 immunoreactivity in the basal and suprabasal layers (arrowheads), while the thickened granular layers in the upper epidermis exhibit normal CALML3 staining including nuclear accumulation (arrows). The images shown are representative of all 5 samples analyzed.

Figure 5 shows CALML3 staining in an example of psoriasis. These more regular lesions with lymphocytic infiltrate, inflammation, and parakeratosis exhibited variable, but overall fairly normal CALML3 expression across the samples tested. Ki-67 also showed variable staining in these samples, indicating different states of (hyper-) proliferation. However, the overall pattern remained consistent with an inverse relationship between Ki-67 and CALML3 staining. CALML3 staining increased in the suprabasal layers of non-proliferating cells where prominent nuclear localization was also observed (Fig. 5).

**Figure 6:**
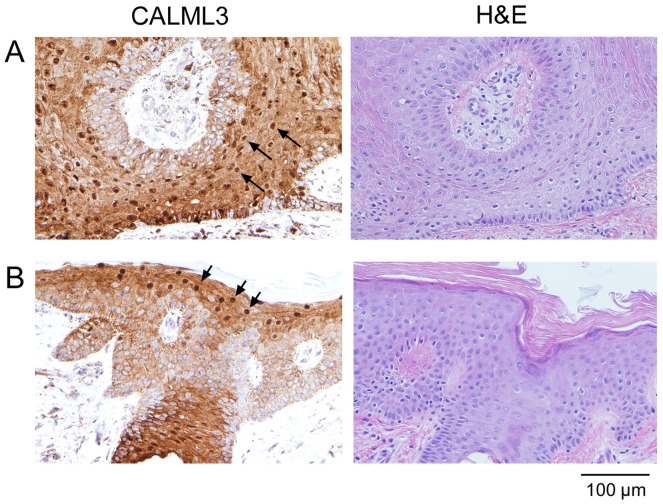
CALML3 immunolocalization in verruca and ichthyosis. Sections from a biopsy specimen of a verruca (A) and ichthyosis (B) were stained for CALML3 and H&E as indicated. CALML3 is robustly expressed in the thickened and irregular epidermal layers of these benign skin lesions, with prominent nuclear accumulation (arrows) in the uppermost granular layers. The images shown in panels A and B are representative of all verruca and ichthyosis samples analyzed, respectively.

CALML3 staining was also investigated in specimens of verruca (warts) and ichthyosis, two additional types of skin lesion characterized by a thickening of the epidermis and keratinocyte hyperproliferation. As shown in Figure 6, CALML3 was robustly expressed in the cytoplasm and periphery of basal and suprabasal cells in the irregular and thickened layers of epidermis, and showed distinct nuclear localization in the uppermost cell layers. These characteristics reflect the expression pattern observed in the normal epidermis (Fig. 1) and thus indicate the well-differentiated nature of these non-malignant skin lesions.

## Discussion

CALML3 expression and localization appear to be regulated during keratinocyte differentiation [8]. Earlier studies indicated that CALML3 is downregulated in breast cancer and absent in most transformed cell lines [2], [9]. We therefore hypothesized that CALML3 may serve as an indicator of normalcy in the human epidermis. In contrast, CALML3 may be aberrantly expressed and/or downregulated in hyperproliferative disorders such as psoriasis and ichthyosis, as well as in pre-cancerous lesions (such as AK) and in squamous cell and basal cell non-melanoma skin cancers. We tested this by immunohistochemistry on biopsies representing different skin disorders. We also compared CALML3 staining with labeling for Ki-67, a known marker of proliferation, since it is thought that CALML3 expression is induced during differentiation of non-proliferating keratinocytes. Although the current study was limited to a total of 50 tissue samples approved for analysis by the IRB protocol, we analyzed a minimum of 5 different patient samples for each skin disorder, which thus provided a representative overview of CALML3 expression and localization in these different conditions.

In the normal human skin, CALML3 was confined to the epidermis and accessory skin organs such as sebaceous glands and hair follicle epithelium, as reported previously [8]. CALML3 staining is weak in the basal layer and increases in intensity in the suprabasal layers extending into the granular layers. In the uppermost layers the cells show strong cytoplasmic and peripheral labeling and are often intensely immunoreactive for CALML3 in the nucleus. In contrast, Ki-67 stains only the basal layer of cells in a narrow line consistent with other studies [12] and the notion that basal (stem) cells are responsible for proliferation and keratinocyte renewal in the epidermis. The inverse relationship between Ki-67 and CALML3 staining was consistent in all tissue samples analyzed and represents a significant finding of this study.

Actinic keratoses are a diverse subset of skin pathologies ranging from relatively normal epidermis with intact and well-differentiated tissue structure to highly disorganized tissue with loss of normal cellular stratification and SCC-like appearance. This was reflected in the increased Ki-67 expression along the basal layer in samples with basal hyperproliferation but otherwise relatively normal appearance (illustrated in Fig. 2B) as compared to the widespread and scattered Ki-67 labeling and loss of polarity in highly proliferating samples (illustrated in Fig. 2C). However, in each case CALML3 expression was reduced and nuclear expression lost in the proliferating regions of the lesion. Since these lesions are often considered pre-cancerous, a reduction of staining and especially a loss of nuclear CALML3 staining indicate that such lesions are less well differentiated and potentially progressing towards an invasive phenotype. Thus, co-staining biopsies for CALML3 and a proliferation marker such as Ki-67 could be of diagnostic value.

CALML3 and Ki-67 staining are also highly informative in SCC and BCC specimens. In all cases CALML3 staining was strongly reduced in the tumors when compared to the overlying epithelium. When residual CALML3 expression was detected in the tumor, it was mostly cytoplasmic and there was a consistent loss of CALML3 labeling in the nucleus. In the tumors, Ki-67 staining tends to be irregular and often without polarity, reflecting pockets of highly proliferating cells. Although double staining for CALML3 and Ki-67 has not yet been systematically performed in other cancers, our results in skin cancer are in good agreement with findings in cervical and prostate carcinoma as well as in breast cancer: In all cases, CALML3 was found to be strongly reduced in the tumors [1], [2]. In cases where the transition from benign hyperplasia to a more invasive phenotype is not readily apparent, more subtle changes in CALML3 expression and localization may be useful as indicators of the status of a given lesion. Of particular interest is the accumulation of CALML3 in the nuclei of fully differentiated keratinocytes in the spinous and granular layers. The absence of nuclear CALML3 staining is one of the most characteristic signs of incomplete or absent terminal differentiation in the epidermis and may thus be an early sign of abnormal development in the epidermis and the progression to a potentially malignant phenotype.

CALML3 staining was also investigated in tissue sections of non-cancerous hyperproliferative skin lesions including verruca, ichthyosis and psoriasis. These are characterized by isolated (verruca) or more diffuse and widespread areas of thickened skin, with psoriasis being an auto-immune disease typified by hyperproliferating epidermis with parakeratosis and lymphocytic infiltrate [13]. CALML3 stained all these samples in a polarized manner similar to normal skin, clearly revealing the expanded and often distorted layers of epidermis. Interestingly, parakeratotic layers also exhibited CALML3 staining and nuclear CALML3 accumulation, suggesting that the cells in these areas of the lesions are well differentiated. On the other hand, as in the cancer specimens, CALML3 expression was always absent in areas of high proliferation.

The function of CALML3 in the epidermis, and the significance of its expression in the nucleus of differentiated keratinocytes in the uppermost layers of the skin remain unknown. Similar to the ubiquitous Ca^2+^ sensor protein calmodulin, CALML3 function is linked to its target proteins. The only bona-fide target protein described so far for CALML3 is the unconventional myosin-10 (Myo10). CALML3 is a specific light chain of Myo10 and increases Myo10 expression and function [3], [4], [14]. Myo10 accumulates at the cell periphery where it is involved in dynamic actin remodeling and cell migration and adhesion [7], [15]. In epidermal wound healing, CALML3-mediated Myo10 function may be required for keratinocyte migration and re-epithelialization [11]. The peripheral staining seen for CALML3 in differentiating keratinocytes thus likely reflects the localization of its target Myo10. On the other hand, the nuclear target(s) of CALML3 has (have) not yet been identified. However, a possible candidate was recently isolated in a yeast two-hybrid screen using CALML3 as bait (RDB and EES, unpublished data). This protein of unknown function, named IQ-containing protein E (IQCE) shows domain similarity to chromosome segregation and DNA repair enzymes and may thus be present in the nucleus. Further work will be needed to characterize the target(s) and regulatory function of CALML3 in the nucleus of differentiated keratinocytes, and to determine the link, if any, between the absence of nuclear CALML3 and failing differentiation in these cells. One intriguing possibility is that the presence of nuclear CALML3, which is typically observed in the uppermost, differentiated layers of the skin is characteristic of pre-apoptotic cells. CALML3 may be required to regulate processes leading to the final, anucleate state of these cells, e.g., by interacting with a nuclear protein involved in chromosome fragmentation.

Because the transition from a benign hyperplastic skin disorder to invasive malignant cancer is often difficult to recognize and may occur over an extended period of time, it is important to develop new methods of screening and detecting de-differentiation, transformation and proliferation of cancerous and pre-cancerous lesions. For example, AK is considered a pre-cancerous lesion but if untreated, can develop into SCC [16]. Whereas BCC rarely metastasizes, SCC can metastasize and result in death if not treated in a timely fashion. Analysis of CALML3 expression status, and particularly its localization at the periphery and in the nucleus of differentiated cells in the epidermis may become a useful tool in the differential diagnosis of non-malignant versus malignant and invasive skin disorders. Because loss of CALML3 expression appears to occur very early in cancer development [2], CALML3 immunostaining may not be particularly useful in staging the progression of existing cancer. On the other hand, the loss of CALML3 staining (and especially the absence of nuclear staining) is highly characteristic of malignant skin cancer. CALML3 immunohistochemistry could thus become a useful diagnostic tool in pathology assessment, providing one of the earliest indicators of malignant development of skin disease and thereby facilitating decision-making prior to surgery. The robust expression of CALML3 in the normal epidermis together with the availability of suitable antibodies for immunohistochemical detection on properly stored biopsies should make this a readily applicable approach for the skin pathologist.
